# Intestinal Microbiome in Hematopoietic Stem Cell Transplantation For Autoimmune Diseases: Considerations and Perspectives on Behalf of Autoimmune Diseases Working Party (ADWP) of the EBMT

**DOI:** 10.3389/fonc.2021.722436

**Published:** 2021-10-22

**Authors:** Tobias Alexander, John A. Snowden, Joachim Burman, Hyun-Dong Chang, Nicoletta Del Papa, Dominique Farge, James O. Lindsay, Florent Malard, Paolo A. Muraro, Rosamaria Nitti, Azucena Salas, Basil Sharrack, Mohamad Mohty, Raffaella Greco

**Affiliations:** ^1^Department of Rheumatology and Clinical Immunology – Charité–Universitätsmedizin Berlin, Corporate Member of Freie Universität Berlin, Humboldt-Universität zu Berlin, and the Berlin Institute of Health (BIH), Berlin, Germany; ^2^Deutsches Rheuma-Forschungszentrum (DRFZ Berlin) – a Leibniz Institute, Berlin, Germany; ^3^Department of Haematology, Sheffield Teaching Hospitals Foundation NHS Trust, Sheffield, United Kingdom; ^4^Department of Oncology and Metabolism, University of Sheffield, Sheffield, United Kingdom; ^5^Department of Neuroscience, Uppsala University, Uppsala, Sweden; ^6^Institute of Biotechnology, Technische Universität Berlin, Berlin, Germany; ^7^Scleroderma Clinic, Dip. Reumatologia, ASST G. Pini-CTO, Milano, Italy; ^8^Unité de Médecine Interne: (UF 04) CRMR MATHEC, Maladies Auto-Immunes et Thérapie Cellulaire, Paris, France; ^9^Universite de Paris, IRSL, Recherche Clinique Appliquee `à l’´hématologie, Paris, France; ^10^Department of Medicine, McGill University, Montreal, QC, Canada; ^11^Centre for Immunobiology, Blizard Institute, Barts and the London School of Medicine, Queen Mary University of London, London, United Kingdom; ^12^Service d’hématologie Clinique et de Thérapie Cellulaire, Hôpital Saint Antoine, APHP, Sorbonne Université, INSERM UMRs 938, Paris, France; ^13^Department of Brain Sciences, Imperial College London, London, United Kingdom; ^14^Unit of Hematology and Bone Marrow Transplantation, IRCCS San Raffaele Scientific Institute, Vita-Salute San Raffaele University, Milan, Italy; ^15^IDIBAPS, CIBER-EHD, Barcelona, Spain; ^16^Department of Neuroscience, Sheffield Teaching Hospitals NHS, Foundation Trust, Sheffield, United Kingdom; ^17^NIHR Neurosciences Biomedical Research Centre, University of Sheffield, Sheffield, United Kingdom

**Keywords:** autoimmune diseases, autoimmunity, fecal transplantation, intestinal, microbiome, stem cell transplantation, HSCT = hematopoietic stem cell transplant

## Abstract

Over the past decades, hematopoietic stem cell transplantation (HSCT) has been evolving as specific treatment for patients with severe and refractory autoimmune diseases (ADs), where mechanistic studies have provided evidence for a profound immune renewal facilitating the observed beneficial responses. The intestinal microbiome plays an important role in host physiology including shaping the immune repertoire. The relationships between intestinal microbiota composition and outcomes after HSCT for hematologic diseases have been identified, particularly for predicting the mortality from infectious and non-infectious causes. Furthermore, therapeutic manipulations of the gut microbiota, such as fecal microbiota transplant (FMT), have emerged as promising therapeutic approaches for restoring the functional and anatomical integrity of the intestinal microbiota post-transplantation. Although changes in the intestinal microbiome have been linked to various ADs, studies investigating the effect of intestinal dysbiosis on HSCT outcomes for ADs are scarce and require further attention. Herein, we describe some of the landmark microbiome studies in HSCT recipients and patients with chronic ADs, and discuss the challenges and opportunities of microbiome research for diagnostic and therapeutic purposes in the context of HSCT for ADs.

## Introduction

Intestinal microbiota may positively affect many aspects of the host physiology, including absorption of nutrients, prevention of overgrowth by potential pathogens, maintenance of epithelial barrier function, and shaping the immune system (1). Studies of the microbiome in the setting of hematopoietic stem cell transplantation (HSCT) demonstrated that intestinal flora are of particular importance in determining treatment outcomes, influencing immune reconstitution, and impacting complications such as infections or graft-versus-host disease (GvHD) (2, 3). In addition, changes in the microbial composition and function have been associated with various autoimmune diseases (ADs), and, although the precise mechanistic links between the microbiome and ADs remain largely unknown, increasing evidence suggests that disturbed gut microbiota contribute to pathogenesis (4). Among the potential mechanisms in the complex interplay between gut microbiota and host immune system, abnormal microbial translocation, molecular mimicry, and dysregulation of local and systemic immunity have been postulated.

This article will summarize the current evidence supporting the relationship between the microbiome and specific ADs, its impact on transplant outcomes, and potential therapeutic interventions, such as fecal microbiota transplantation (FMT). Moving forward, we propose how we may evaluate and influence the microbiome in the setting of HSCT for ADs to affect immune reconstitution and potentially improve clinical outcomes.

## Interaction Between Gut Microbiota and the Host Immune System

**Figure 1:**
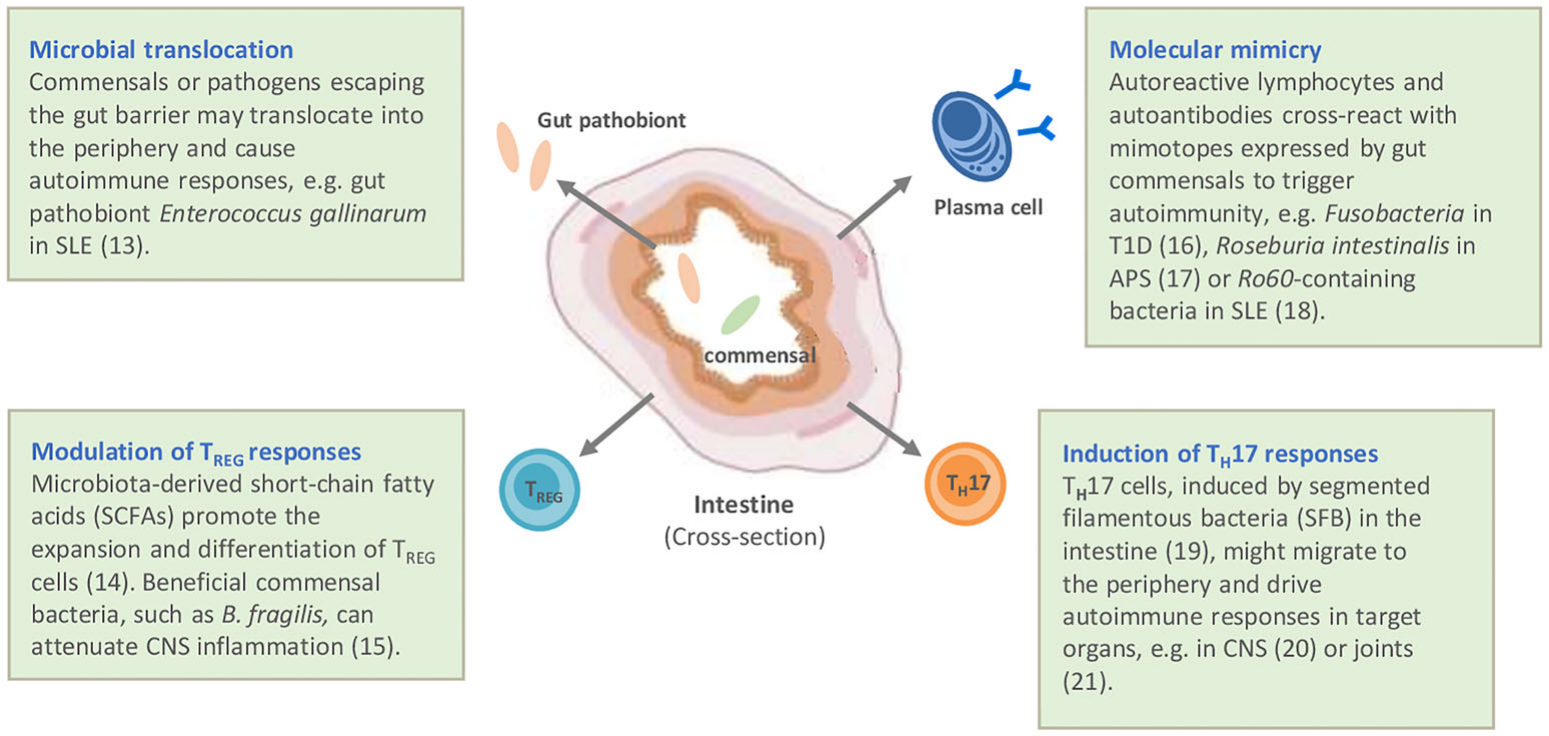
Potential mechanisms by which autoimmune diseases are linked to gut microbiota and intestinal immunity. Modified according to (12). APS, antiphospholipid syndrome; CNS, central nervous system; SLE, systemic lupus erythematosus; T1D, type I diabetes (13–21).

While the primary function of the intestinal microbiota for the host has been considered to be the digestion of complex sugars and the provision of essential vitamins, it has become clear that the microbiota play an important role in the education and shaping of a functioning immune system. Evidence for this comes from the analysis of germ-free mice, which in the absence of any microbiota have underdeveloped lymph organs and reduced innate immune competence resulting in increased susceptibility to infection (5). Most likely for similar reasons, germ-free mice are resistant to genetic and induced models of autoimmunity. While the molecular mechanisms are still poorly understood, several pathways involved in the microbiota–host interaction have been identified, ranging from provision of ligands for innate receptors, such as Toll-like receptors for “trained” immunity (6), to the production of short-chain fatty acids, a product of the metabolizing of dietary fibers by certain bacteria, which have been described to enhance immune regulation (7, 8). Reciprocally, the host controls the microbiota through the production of antimicrobial peptides by intestinal epithelial cells and copious amounts of IgA antibodies, which are actively transported into the gut lumen by the intestinal epithelial cells, controlling the growth, mobility and attachment of intestinal bacteria (9). Alterations to this intricate microbiota – host interaction, e.g. genetic defects disrupting microbial sensing of the host or loss of bacterial diversity, often summarized under the term *dysbiosis*, resulting in loss of microbial functions for the host, has been associated with the development of chronic inflammatory diseases (10). Mechanistically, several pathways have been discussed by which intestinal microbiota might contribute to the development or perpetuation of autoimmune diseases (11). They include gut dysbiosis, which disrupts local gut homeostasis and may promote translocation of commensal or pathobionts to tissues where they facilitate chronic inflammation. In addition, microbiota may trigger autoimmunity directly by providing antigenic stimuli resulting in cross-reactivity of autoreactive lymphocytes and autoantibodies with bacterial orthologues. Finally, microbiota may modulate the immune system through their metabolites and may facilitate immune regulation by stimulating regulatory immune elements (summarized in **Figure 1**).

## Microbial Profiling

The introduction of molecular biological methods for the characterization of the microbiota, in particular high-throughput sequencing, has greatly advanced our understanding of the diversity and function of the microbiota (22). Sequencing of the single or a combination of the 9 variable regions of the gene for the 16S ribosomal RNA of the small 30S ribosomal subunit is the mainstay to describe the composition of a microbial community. While 16S rRNA sequencing has become the method of choice due to its simplicity, it is often limited in the taxonomic resolution and is prone to bias e.g. PCR amplification and sampling depth (23). More extensive sequencing approaches include whole 16S rRNA gene sequencing allowing resolution of the microbiome to the species level, however often at the cost of sampling depth, and shot-gun metagenomics sequencing which will additionally yield information on the genetic repertoire, i.e. potential functional genes, of the bacterial community, the latter requiring extensive bioinformatics resources. Other “omics”, such as metaproteomics can also be used to define the composition as well as the function of the microbiota, while metabolomic profiling identifies the mediators with which the microbiota could interact within itself and with the host [reviewed in (24)]. Recently, the combination of absolute quantification of the microbiota by flow cytometry with 16S rRNA gene profiling was shown to better reflect clinically relevant changes of the microbiome in patients with inflammatory bowel diseases (IBD) (25). Flow cytometric analysis of the microbiota also has the potential to rapidly identify alterations in the microbiota on the single cell level for monitoring purposes (26) and when combined with cell sorting and 16S rRNA gene analysis could lead to the identification of relevant bacteria in a more targeted fashion.

## Role of Intestinal Microbiota in Autoimmune Diseases

### Inflammatory Bowel Diseases

The intestinal tract, home to the largest density and diversity of microorganisms in healthy humans, is the target organ of IBD comprising Crohn’s disease (CD) and Ulcerative Colitis (UC). The chronic intestinal inflammation in IBD is characterized by effector and tissue resident memory T cell responses to aspects of the intestinal microbiota (27). Despite large inter-individual variability, genetic analyses of microbial populations in stool and/or mucosal biopsies has revealed an overall decrease in diversity, a loss of symbionts and an increase in pathobionts (essentially Gram-negative proinflammatory microbes) in both UC and CD (27, 28). Whether these changes in microbial composition and IBD pathogenesis are a cause or consequence of intestinal inflammation remains a key area of study (29). Alterations in gut microbiota can disrupt epithelial and immune homeostasis, leading to increased permeability and eventual immune activation. Alternatively, the documented genetic and/or microbial-independent environmental factors associated with IBD may promote inflammation and oxidative stress, which subsequently results in a shift in microbial composition. A recent study has shown that increased fecal proteolytic activity and microbiota changes precede diagnoses of ulcerative colitis (30). In addition, the altered humoral and cellular acquired immune responses towards bacterial antigens that characterize IBD, particularly Crohn’s disease (31), may predate disease onset (32). This suggests that immune responses towards microbes, rather than microbial composition itself, drives epithelial barrier disruption and altered innate responses at disease onset. Thus, in marked contrast to the impressive efficacy seen in Clostridium difficile infection (33), FMT has shown some benefit in mild UC but no impact in CD (34–36). Nonetheless, regardless of whether dysbiosis is the initial event or the result of overt inflammation, shifts in microbial composition may help perpetuate disease, as well as impact response to therapy in IBD (37, 38), and thus represent a desirable target for future therapies.

### Systemic Sclerosis

In systemic sclerosis (SSc), a rare systemic autoimmune disease characterized by vasculopathy, immune activation and consequent progressive fibrosis, multiple genetic, epigenetic, and environmental factors are regarded as potential triggers for the onset and progression of the disease (39). Over the past decades, emerging evidence suggests that alterations of microbial populations colonizing epithelial surfaces (i.e., gastrointestinal tract, skin and lung), known as dysbiosis, may contribute to chronic inflammation and autoimmunity (4). Since the gastrointestinal tract is one of the organs highly affected in SSc, recent studies have aimed to investigate gastrointestinal microbiota alterations to elucidate the possible interaction with disease phenotype and clinical outcome of the disease (40). Initial studies found that specific bacteria, particularly beneficial commensal genera (*Faecalibacterium, Clostridium* and *Rikenella*) and, conversely, more potentially pathobiont genera (*Bifidobacterium, Fusobacterium* and *Prevotella*) were decreased in SSc patients compared to healthy controls (41–43). Notably, SSc patients with more severe gastrointestinal symptoms exhibited a prevalence of the pathobiont *Fusobacterium* compared to patients with mild or no gastrointestinal symptoms (41–43). Furthermore, overabundance of opportunistic pathogenic *Clostridium* and typically oral *Streptococcus* species was recently described in SSc, while *Alistipes*, *Bacteroides*, and butyrate-producing species were depleted, congruent with findings in patients with IgG4-related disease, suggesting a common signature in both fibrosis-prone autoimmune diseases (44). Altogether, these studies confirm the existence of a shift in gut microbiota population in SSc patients. Whether these changes are causative or rather reflect the gastrointestinal involvement by inflammatory and fibrotic processes remains to be demonstrated. The role of intestinal dysbiosis in the disease pathogenesis is further complicated by the possibility that, as showed in other diseases, the intestinal microbiota in SSc might modulate local immunological mechanisms possibly responsible of local and systemic alterations (45).

### Multiple Sclerosis

Multiple sclerosis (MS) is a chronic immune-mediated disease of the central nervous system (CNS), which results from interactions of genetic and environmental factors (46). The underlying pathological process is complex but includes the abnormal activation of T and B cells targeting foreign and/or self-antigens, which could be primed within the CNS or in the periphery (47, 48). A potential source of such antigens is the gut microbiome, which exhibits a level of homology to human myelin proteins and may trigger cross-reactivity through the mechanism of ‘molecular mimicry’ (49, 50). Immune reconstitution studies have shown that gut Mucosal Associated Invariant T (MAIT) cells, which express chemokine receptor 6 (CCR6) to facilitate their transmigration into the CNS, are reduced following autologous HSCT, suggesting that they may play a role in crosslinking gut microbiome with the neuroaxis (51). In one experimental model, the presence of intestinal microbiota was necessary to induce CNS autoimmunity, suggesting that the gut has the ability to control systemic autoimmune responses (52, 53). Germ-free mice recipients receiving feces from patients with MS develop severe Experimental Allergic Encephalomyelitis (EAE) and the administration of *Lactobacilli* seems to suppress this process (54, 55). A number of case control studies have reported reduced gut microbiome diversity in patients with MS, particularly in those with active disease, although a consistent microbiome phenotype has not been identified (56, 57). Furthermore, some MS orally administered disease modifying therapies have been found to inhibit the growth of *Clostridium in vitro*, which may contribute to their anti-inflammatory mechanism of action (58). Given the increasing evidence that gut microbiome plays a role in the immune system homeostasis and in the pathogenesis of MS, changes in the microbiome in patients with MS undergoing HSCT warrant investigation.

## Role of Intestinal Microbiota in Hematopoietic Stem Cell Transplantation

### Correlation With HSCT Outcomes

The intestinal microbiome undergoes profound changes during the course of transplantation. Multiple transplant-related factors (i.e. conditioning regimen, broad-spectrum antibiotics, nutrition) drive microbial shifts. At the same time, the alteration in the composition of gut flora is associated with transplant outcomes, including overall survival (OS), progression-free survival (PFS), treatment-related mortality (TRM) and GvHD (**Table 1**). Bacterial diversity largely decreases after HSCT, and is correlated with increased risk of major transplant complications such as infections or GvHD, potentially affecting the outcome of the procedure (81, 82). A large multicenter observational study has confirmed lower mortality rates in patients showing higher diversity of intestinal microbiota at engraftment (3). Recently, microbiota injury has been observed also in recipients of autologous HSCT, who undergo similar antibiotic exposures and nutritional alterations after high-dose chemotherapy and transplant procedure (80). Reduced OS and PFS have been reported in patients with lower peri-engraftment microbiome diversity.

**Table 1:** Impact of microbiome on HSCT outcomes.

Study	Study Population	Microbiome Analysis	Microbiome Biomarker	HSCT Outcome
Taur et al., 2012 (59)	94 adult patients Allogeneic HSCT Single center, USA	454 pyrosequencing, V1-V3 region of the 16S RNA gene	Enterococcus domination (>30%)	VRE Bacteremia 9-fold increased risk
			Proteobacteria domination (>30%)	Gram negative Bacteremia 5-fold increased risk
Ubeda et al., 2013 (60)	94 adult patients Allogeneic HSCT Single center, USA	454 pyrosequencing, V1-V3 region of the 16S RNA gene	Barnesiella genus* enteric colonization	Protection from VRE domination
Taur et al., 2014 (61)	80 adult patients Allogeneic HSCT Single center, USA	454 pyrosequencing, V1-V3 region of the 16S RNA gene	Low bacterial diversity at engraftment	Lower OS Higher TRM
Holler et al., 2014 (62)	31 adult patients Allogeneic HSCT Single center, Germany	Roche 454 platform sequencing, V3 region of the 16S RNA gene Strain-specific PCR of enterococci Urinary indoxyl sulfate analysis **	Enterococcus abundance > 20%	Increased frequency of GI acute GvHD
			Urinary indoxyl sulfate levels decrease during aplasia after HSCT	–
Weber et al., 2015 (63)	131 adult patients Allogeneic HSCT Single center, Germany	Roche 454 platform sequencing, V3 region of 16S RNA gene Strain-specific PCR of enterococci Urinary indoxyl sulfate analysis **	Low urinary indoxyl sulfate levels within day +10 after HSCT (Lachnospiraceae and Ruminococcaceae *** » high urinary indoxyl sulfate levels; Bacilli » low indoxyl sulfate levels)	Low OS High TRM
Jenq et al., 2015 (64)	115 adult patients Allogeneic HSCT Single center, USA	First cohort (n=64): Roche 454 platform sequencing, V1-V3 region of the 16S RNA gene Second cohort (n=51): Illumina MiSeq platform sequencing, V4-V5 region of the 16S RNA gene	Increased bacterial diversity	Higher OS Lower TRM Lower GvHD related mortality
			Blautia genus # abundance	Higher OS Lower GvHD related mortality Lower incidence of acute GvHD requiring systemic corticosteroids or steroid-refractory
			Lachnospiraceae abundance Clostridiales abundance Clostridia abundance	Lower GvHD related mortality
Shono et al., 2016 (65)	857 adult patients Allogeneic HSCT Single center, USA	Illumina MiSeq platform sequencing, V4-V5 region of the 16S RNA gene	Imipenem-cilastatin treatment Piperacillin-tazobactam treatment (associated to loss of Bacteroidetes and Lactobacillus ##)	Higher GvHD related mortality Higher grades 2-4 acute GvHD Higher GI acute GvHD
Harris et al., 2016 (66)	94 adult patients Allogeneic HSCT Single center, USA	454 pyrosequencing, V1-V3 region of the 16S RNA gene	Low baseline diversity Enterococcus domination (>30%)	Higher risk of pre-engraftment pulmonary complications
			γ-Proteobacteria domination (>30%)	Higher risk of post-engraftment pulmonary complications
Peled et al., 2017 (67)	541 adult patients Allogeneic HSCT Single center, USA	Illumina MiSeq platform sequencing, V4-V5 region of the 16S RNA gene	Abundance of Eubacterium limosum and other related bacteria	Lower relapse/progression of disease risk
Mancini et al., 2017 (68)	96 adult patients Allogeneic HSCT Single center, Italy	Roche 454 platform sequencing, V3-V5 region of the 16S RNA gene	Baseline Enterobacteriaceae >5%	Higher risk of microbiologically confirmed sepsis, severe sepsis and septic shock
			Baseline Lachnospiraceae ≤10%	Lower OS Higher infectious related mortality Higher non-infectious related mortality
Doki et al., 2017 (69)	107 adult patients Allogeneic HSCT Single center, Japan	Roche 454 platform sequencing, V1–2 region of the 16S RNA gene	Higher abundance of Firmicutes, lower abundance of Bacteroidetes, higher abundance Fecal bacterium and Eubacterium at baseline	Higher risk of acute GvHD
Lee et al., 2017 (70)	234 adult patients Allogeneic HSCT Single center, USA	Illumina MiSeq platform sequencing, V4-V5 region of the 16S RNA gene	Combined abundance of Bacteroidetes phylum, Lachnospiraceae family, Ruminococcaceae family	Protection from Clostridium difficile infection
			Enterococcus faecalis at various rank designations	Higher risk of Clostridium difficile infection
Golob et al., 2017 (71)	66 adult patients Allogeneic HSCT Single center, USA	Illumina MiSeq platform sequencing, V3-V4 region of the 16S RNA gene	Presence of oral Actinobacteria and oral Firmicutes in stool, deficit of Lachnospiraceae at neutrophil engraftment	Higher risk of acute GvHD
Stein-Thoeringer et al., 2019 (72)	1325 adult patients Allogeneic HSCT Four centers: USA, Germany, Japan	Illumina MiSeq platform sequencing, V4-V5 region of the 16S RNA gene	Enterococcus domination (>30%) at early post-transplant period (day 0 to day +12)	Lower OS Higher GvHD related mortality Higher grades 2-4 acute GvHD incidence
Galloway-Peña et al., 2019 (73)	44 adult patients Allogeneic HSCT Single center, USA	Illumina MiSeq platform sequencing, V4 region of the 16S RNA gene	Low microbial diversity at engraftment	Higher risk of intestinal acute GvHD Higher TRM
			Low Coriobacteriia, Coriobacteriaceae at engraftment	Higher risk of intestinal acute GvHD
Biagi et al., 2019 (74)	36 pediatric patients Allogeneic HSCT Four centers, Italy	Illumina MiSeq platform sequencing, V3-V4 region of the 16S RNA gene	Pretransplant Blautia genus abundance	Lower acute GvHD risk
			Pretransplant Fusobacterium abundance	Higher severe GI acute GvHD risk
			Abundance of Bacteroides at engraftment	Higher grades 2-4 acute GvHD risk
Han et al., 2019 (75)	141 adult patients Allogeneic HSCT China	Illumina MiSeq platform sequencing, V3-V4 region of the 16S RNA gene	At day 15 after HSCT: Low diversity Low Lachnospiraceae Low Peptostreptococcaceae Low Erysipelotrichaceae High Enterobacteriaceae	Higher acute GvHD risk Higher acute GvHD grades
Lee et al., 2019 (76)	211 adult patients Allogeneic HSCT Single center, Korea	16S rRNA gene sequencing	Post engraftment: Loss of diversity compared to pre transplant sample Depletion Ruminococcus Increase of Eubacterium Increase of Escherichia	Higher risk of intestinal acute GvHD
Peled et al., 2020 (3)	1362 adult patients Allogeneic HSCT Four centers: USA, Germany, Japan	Illumina MiSeq platform sequencing, V4-V5 region of the 16S RNA gene	Higher intestinal diversity in the peri-engraftment period (between days 7 and 21 after HSCT)	Higher OS Lower TRM Lower GvHD related mortality §
			Higher intestinal diversity before HSCT (from day –30 to –6)	Higher OS Lower TRM
Payen et al., 2020 (77)	70 adult patients (n=35 with GvHD; n=35 without GvHD) Allogeneic HSCT Single center, France	Illumina MiSeq platform sequencing, V3-V4 region of the 16S RNA gene	Lower microbial diversity Depletion of Blautia Reduction of Lachnospiraceae and Ruminococcaceae Increase of Prevotella and Stenotrophomonas §§	Severe acute GvHD
Han et al., 2020 (78)	150 adult patients Allogeneic HSCT Two centers, China	Illumina MiSeq platform sequencing, V3-V4 region of the 16S RNA gene	Gut microbiota score: a formula based on selected gut microbiota features	Risk of grades 2-4 acute GvHD
Greco et al., 2021 (79)	96 adult patients Allogeneic HSCT Single center, Italy	Roche 454 platform sequencing, V3-V5 region of the 16S RNA gene	Low Diversity at day +10 after HSCT	Higher grades 2-4 acute GvHD Higher grades 3-4 acute GvHD Higher risk of GI involvement Higher risk of acute GvHD with skin involvement
			Enterococcaceae > 90% at day +10	Higher grades 2-4 acute GvHD Higher grades 3-4 acute GvHD Higher risk of acute GvHD with GI involvement
			<10% Lachnospiraceae at day +10	Higher risk of acute GvHD with GI involvement
			Staphylococcaceae >40% at day +10	Higher risk of acute GvHD with GI involvement Higher risk of acute GvHD with liver involvement Higher risk of steroid-refractory acute GvHD
Khan et al., 2021 (80)	534 adult patients Autologous HSCT Two centers, USA	Illumina MiSeq platform sequencing, V4-V5 region of the 16S RNA gene	Increased bacterial diversity at peri-neutrophil engraftment period	Higher PFS
			Post-engraftment increased bacterial diversity	Higher PFS and OS
			Abundance of Enterococcus	Lower OS

*Barnesiella genus belongs to the family Porphyromonadaceae, within the phylum Bacteroidetes.

**Urinary indoxyl sulfate originates from the degradation of tryptophan to indole by colonic microbiota, followed by microsomal oxidation to indoxyl and sulfonation.

***Families of Lachnospiraceae and Ruminococcaceae belong to the class of Clostridia, phylum Firmicutes. Eubacterium rectale is a prominent member of the family of Lachnospiraceae.

#Blautia genus is classified as follows: family Lachnospiraceae, order Clostridiales, class Clostridia, and phylum Firmicutes.

##This study analyzed antibiotic treatment impact on GvHD risk, then antibiotic impact on microbiome within the same population.

§GvHD related mortality was significantly lower in patients with higher intestinal diversity in transplant from unmanipulated grafts.

§§Prevotella and Stenotrophomonas respectively belong to the Bacteroidetes and Proteobacteria families.

GvHD, Graft-versus-Host Disease; GI, gastrointestinal; HSCT, Hematopoietic Stem Cell Transplantation; OS, Overall Survival; PFS, Progression-Free survival; TRM, Transplant-related mortality; VRE, Vancomycin-resistant Enterococcus.

### Impact of chemotherapy, Diet, and Antibiotics on the Intestinal Microbiome in Transplant Recipients

Microbiome and transplant correlations may be influenced by local practices, antibiotic choices, hospital flora, and diet. Gastrointestinal disturbances associated with chemotherapy and radiation (83) and subsequent mucositis can also impact the composition of intestinal microbiota. A reduction in α-diversity and significant differences in the composition of the intestinal microbiota have been observed in response to chemotherapy, such as increase in *Bacteroides* and *Enterobacteriaceae* paralleled by a decrease in *Bifidobacterium*, *Faecalibacterium prausnitzii*, and *Clostridium cluster XIVa* (84), and a drastic drop in *Faecalibacterium* accompanied by an increase of *Escherichia* (85). The impact of diet on gut flora is well-recognized (86). Depletion of the intestinal microbiota reduces visceral adipose tissue and caloric uptake from diet (87), and enteral feeding may exert a beneficial effect on intestinal flora by providing the required nutrients (88). Interestingly, a lactose-free diet can prevent microbial overdominance by detrimental commensal bacteria like *Enterococcus* (72). Broad-spectrum antibiotic prophylaxis/treatment, commonly used in HSCT recipients, in the early phase after HSCT can beneficially reduce the number of transmigrated bacteria. However, their long-term effects are detrimental, because they limit microbiota diversity, by killing beneficial commensal bacteria that inhibit pathogens and promote immune defenses (81). A drastic decrease in the diversity of enteric microbiome after administration of antibiotic therapy, and the loss of obligate anaerobic commensal bacteria such as *Clostridia* and *Bacteroidetes* after piperacillin-tazobactam and meropenem administration, are recurrent in literature (89). Metronidazole administration increases enterococcal domination, whereas fluoroquinolone administration reduces domination by *Proteobacteria* (59) and represents an important variable associated with overall survival (61). Broad spectrum antibiotics, by inducing loss of bacterial diversity, are also associated with increased GvHD-related mortality (65, 90).

### Intestinal Microbiome, Immune Reconstitution, and Infection Prevention

Effective and appropriate immune reconstitution is central to successful HSCT. Microbiota populations may influence immune reconstitution and cell dynamics in humans (91). The depletion of the intestinal microbiota impairs post-transplant immune reconstitution (87). Analysis of daily changes in circulating immune cell counts and extended longitudinal microbiota analysis revealed consistent associations between gut bacteria and immune cell dynamics, paving the way for potential microbiota-targeted interventions to improve immunotherapy and treatments for immune-mediated diseases (91).

The gut microbiota play a critical role in maintaining colonization resistance against intestinal pathogens, thus preventing infections. Domination by *Enterococcus* and *Proteobacteria* are associated with the risk of bacteremia by Vancomycin-resistant *Enterococcus* and gram-negative rod respectively (59). A different baseline distribution of the gut microbiome (68) has been reported in patients at risk for microbiologically confirmed infection (high level of *Enterobacteriaceae*, low level of *Lachnospiraceae*), sepsis and septic shock (high level of *Enterobacteriaceae*). Moreover, a documented bloodstream infection may be anticipated by expansion and dominance of pathogenic strains in the gut flora (59, 68, 92, 93). Overall, a low diversity of the intestinal microbiota at engraftment has been shown to be an independent predictor of TRM from both infectious and non-infectious causes (61).

### Intestinal Microbiome, GvHD, and Immunosurveillance

In the allogeneic transplant setting, a regulatory effect of the gut microbiota in the maintenance of intestinal homeostasis has been reported (94). Loss of fecal diversity, as well as increased abundance of members from *Enterococcus* or *Staphylococcus* species have been associated with the incidence and severity of acute GvHD (79), while other organisms such as *Blautia* species have a protective role (64). Metabolites produced by intestinal bacteria may promote intestinal tissue homeostasis and immune tolerance in the context of acute GvHD (95). Moreover, commensal bacteria can also play a role in tumor immunosurveillance. Increased abundance of a cluster of related bacteria including *Eubacterium limosum* was associated with decreased risk of relapse or disease-progression (67).

Altogether, these results indicate that the intestinal microbiota represent a potentially important factor in the success or failure of HSCT. As such, the microbiome can be envisioned both as a biomarker for the identification of patients at higher risk for transplant-related complications, and also a target for intervention aiming to impact clinical outcomes through enhancing microbiota recovery (96).

### Modulation of Gut Microbiota by Fecal Microbiota Transplantation

FMT is a recommended therapeutic strategy for treating recurrent *Clostridioides difficile* infection (97, 98). Additionally, FMT has been investigated for treatment of steroid-resistant acute GvHD and initial positive results (99) were confirmed by several case reports (100). A small cohort study recently reported a complete response in 10 out of 14 patients (71%) with steroid-refractory or steroid-dependent acute GvHD 28 days after FMT (101). This response was accompanied by an increase in microbial α-diversity, a partial engraftment of donor bacterial species, and increased abundance of butyrate-producing bacteria, including groups in the order *Clostridiales*, namely *Blautia* species. Malard et al. recently reported the use of a next-generation FMT product “MaaT013”, a standardized, pooled-donor, high-richness microbiota biotherapeutic, in the largest cohort of patients to date (n=29) with steroid-refractory or steroid-dependent intestinal acute GvHD (102). These patients had previously received and failed 1 to 5 lines of GvHD systemic treatments. The product was well tolerated and at day 28, overall response and complete remission rates were 59% and 31%, respectively. Furthermore, some studies have evaluated the role of FMT in treating dysbiosis after allogeneic HSCT. Taur et al. reported that autologous FMT after HSCT was safe and boosted microbial diversity, restoring bacterial populations lost during HSCT and reversing the disruptive effects of the broad-spectrum antibiotics (n=14) (81). Overall, FMT appears to be a promising strategy and several studies are ongoing to evaluate FMT for acute GvHD management (NCT03812705, NCGT03492502, NCT03359980, NCT03720392, NCT03678493). Regarding prevention of complications, additional studies are warranted to confirm that restoration of gut microbiota dysbiosis after FMT translates into clinical improvement after allogeneic HSCT, in particular a lower incidence of acute GvHD (96).

## Discussion

It is increasingly accepted that understanding the complex interactions between the microbiome and immune system will be crucial to defining the pathogenesis of ADs, whilst optimizing therapeutic interventions and clinical outcomes. HSCT is increasingly used specifically to treat severe, resistant ADs, with now more than 3000 cases being reported to the registry of the European Society of Bone and Marrow Transplantation (EBMT) (103, 104). To date very limited data is available regarding microbiome biology in the setting of HSCT for ADs, where medium to long-term clinical outcomes are considered to be due to the induction of altered (or ‘re-booted’) immune reconstitution post-transplant. The ‘immune re-boot’ has been increasingly characterized in a range of ADs with a range of immunological markers, including evidence of generation of ‘re-educated’ and regulatory populations to support re-induction of self-tolerance lasting beyond the broad immunosuppressive effects of autologous HSCT (105, 106). Changes in immune reconstitution may affect not only on disease activity, but also adverse events, such as secondary ADs (107–110).

As for ADs outside the transplant setting, and for GvHD in allogeneic HSCT, the microbiome may significantly influence the baseline status of the underlying AD *pre-transplant*, the patients general condition *peri-transplant* (which will inevitably be influenced by the treatment and supportive care, especially antibiotics), and then the dynamics of the reconstituting immune system *post-transplant*. The microbiome may therefore influence short- and long-term immune recovery and clinical outcomes following autologous HSCT. Therefore, future investigations evaluating microbiome changes pre-, peri- and post-HSCT in ADs patients are warranted. **Table 2** includes proposed recommendations for studies of the microbiome (111–115) that could be compared with clinical outcome and laboratory data related to immune reconstitution in patients undergoing HSCT for various ADs. Although bio-banking and testing cannot be regarded as routine care, they could be integrated into clinical trials or observational studies with appropriate institutional approvals. In future, a greater understanding may help design of prospective studies of interventions, including FMT, to test the proof of principle of modulation of the microbiome in this setting.

**Table 2:** Considerations for the analysis of intestinal microbiome in AD undergoing HSCT.

Summary of considerations
Standardization of the microbiome field is complex. Proper documentation of sample collection, data processing, and analysis methods is crucial to be reproducible. The choice of method may also vary, depending on the research interests, simplicity of fecal collection procedures and presence of adequate biobanking infrastructure (63, 66).
*Optimal time-points for sample collection before and after HSCT*: pre-mobilization (usually cyclophosphamide and G-CSF) pre-transplant conditioning (up to 15 days before starting the conditioning regimen) peri-engraftment, i.e. within 7 days following stem cell engraftment Serial post-transplant samples at time points where other immune reconstitution samples are taken (e.g. 3 monthly in the first year, and yearly thereafter, in remission or with stable disease), in the event of relapse and/or progression
*Collection and storage of fecal samples*: Freshly isolated fecal samples, instantly frozen at -80°C without additives (16S rRNA, flow cytometry), widely regarded as the gold standard (69). Samples can be also preserved at −20°C within 15 min after collection, then transferred to a laboratory on dry ice within 24 h of collection and stored at −80°C thereafter (70). Sample collection in tubes containing a DNA stabilizer (e.g. OMNIgene GUT tubes or Stratec stool collection tubes) or 95% ethanol, which allows sample storage at room temperature (16S rRNA) (63, 66).
*Methods of detection*: 16S rRNA sequencing Shot-gun metagenomics sequencing Metabolic profiling Flow cytometric analysis The selection of sequencing methods depends on the scientific questions and sample types: Amplicon sequencing: taxonomic composition of microbiota, cost effective, feasible for large-scale research. Shot-gut Metagenomic sequencing: more information, more expensive than amplicon sequencing. The integration of different methods is advisable, as multi-omics provides insights into both the taxonomy and function of the microbiome (71).
*Bioinformatics analysis*: Several popular software or pipelines are available for data analysis; QIIME and USEARCH are the most largely adopted (71).

AD, autoimmune diseases; HSCT, Hematopoietic Stem Cell Transplantation; G-CSF, granulocyte colony-stimulating factor; FACS, Fluorescence-activated cell sorting.

In conclusion, we have summarized the current evidence supporting the relationship between the microbiome, HSCT and ADs, and speculated on the potential impact of the microbiome on clinical outcomes and immune reconstitution following HSCT for severe, resistant ADs. The evidence in this specific field is currently very limited, warranting harmonization of the microbiome monitoring and prospective studies to evaluate properly any potential impact and/or clinical benefit.

## Data Availability Statement

The original contributions presented in the study are included in the article/supplementary material. Further inquiries can be directed to the corresponding authors.

## Author Contributions

TA, RG, and JS led on concept and design. TA and RG led on coordination and data analysis, provided expert and analytical feedback and were involved in reviewing, writing and editing the manuscript. All authors contributed to the analysis and interpretation of data, and writing sections of the manuscript. The experts on this panel are active members of the EBMT. All co-authors were involved in drafting the paper, revising it critically, and approval of the submitted and final versions.

## Funding

The EBMT provided resources *via* the working party, data office and registry. H-DC is supported by the Dr. Rolf M. Schwiete Foundation and the German Research Foundation through CRC TRR241 B03.

## Conflict of Interest

AS reports research grants from Roche-Genentech, Abbvie, GSK, Scipher Medicine, Alimentiv, Inc, Boehringer Ingelheim and Origo Biopharma; consulting fees from Genentech, GSK, Pfizer, HotSpot Therapeutics, Surrozen, Alimentiv, Origo Biopharma and Morphic Therapeutic. FM reports lecture honoraria from Therakos/Mallinckrodt, Janssen, Biocodex, Sanofi, JAZZ pharmaceuticals and Astellas, all outside the submitted work. MM reports grants and lecture honoraria from Janssen, Sanofi, and JAZZ pharmaceuticals, lecture honoraria from Celgene, Amgen, BMS, Takeda, and Pfizer, consultancy honoraria from MaaT Pharma and grants from Roche, all outside the submitted work. JS declares honoraria for speaking at educational events supported by Jazz, Mallinckrodt, Gilead, Janssen, and Actelion, advisory board participation for Medac, and IDMC membership for a Kiadis Pharma supported clinical trial, none of which are directly related to this review. PM discloses travel support and speaker honoraria from unrestricted educational activities organized by Novartis, Bayer HealthCare, Bayer Pharma, Biogen Idec, Merck-Serono and Sanofi Aventis; and consulting to Magenta Therapeutics and Jasper Therapeutics. RG discloses honoraria for speaking from educational events supported by Biotest, Pfizer, and Magenta. TA received financial support from Amgen, Janssen, Neovii and Mallinckrodt.

The remaining authors declare that the research was conducted in the absence of any commercial or financial relationships that could be construed as a potential conflict of interest.

## Publisher’s Note

All claims expressed in this article are solely those of the authors and do not necessarily represent those of their affiliated organizations, or those of the publisher, the editors and the reviewers. Any product that may be evaluated in this article, or claim that may be made by its manufacturer, is not guaranteed or endorsed by the publisher.
